# Porous Silicon-Based Aptasensors: The Next Generation of Label-Free Devices for Health Monitoring

**DOI:** 10.3390/molecules24122216

**Published:** 2019-06-13

**Authors:** Monica Terracciano, Ilaria Rea, Nicola Borbone, Rosalba Moretta, Giorgia Oliviero, Gennaro Piccialli, Luca De Stefano

**Affiliations:** 1 Institute for Microelectronics and Microsystems, Via P. Castellino 111, 80131 Naples, Italy; ilaria.rea@na.imm.cnr.it (I.R.); rosalba.moretta@na.imm.cnr.it (R.M.); 2 Department of Pharmacy, University of Naples Federico II, Via Domenico Montesano 49, 80131 Naples, Italy; nicola.borbone@unina.it (N.B.); picciall@unina.it (G.P.); 3 Department of Chemical Sciences, University of Naples Federico II, Via Cynthia, 80126 Naples, Italy; 4 Department of Molecular Medicine and Medical Biotechnologies, University of Naples Federico II, Via S. Pansini 5, 80131 Naples, Italy; golivier@unina.it

**Keywords:** aptasensor, porous silicon, surface modification, aptamer, optical label free-sensing

## Abstract

Aptamers are artificial nucleic acid ligands identified and obtained from combinatorial libraries of synthetic nucleic acids through the in vitro process SELEX (systematic evolution of ligands by exponential enrichment). Aptamers are able to bind an ample range of non-nucleic acid targets with great specificity and affinity. Devices based on aptamers as bio-recognition elements open up a new generation of biosensors called aptasensors. This review focuses on some recent achievements in the design of advanced label-free optical aptasensors using porous silicon (PSi) as a transducer surface for the detection of pathogenic microorganisms and diagnostic molecules with high sensitivity, reliability and low limit of detection (LoD).

## 1. Introduction

Biosensors are analytical hybrid devices comprising a bio-receptor (also called bioprobe) immobilized on a transducer surface which is able to selectively recognize a molecular target [1,2]. The most commonly employed bioprobes in biosensors development are enzymes, proteins, and antibodies (Abs), which suffer from various drawbacks, thus limiting their specific applications [3,4]. The enzyme isolation procedure and consequent incorporation in in vitro operating environments could result in a loss of their activity [5]. The Abs are produced by the immune system in response to exposure to antigens, i.e., spores, bacterial toxins, and other foreign substrates. Since Abs are difficult to graft with a proper orientation, i.e., keeping their natural tridimensional structure, sometimes they are less effective in antigen recognition [6]. Nowadays, new biomolecular recognition elements which are able to overcome the Ab and enzyme constraints associated with standard bioprobes are in growing demand in molecular sensing. In this context, aptamers are considered as to have the greatest potential among the recognition tools which have developed in recent years. Aptamers are a class of single-stranded RNA or DNA oligonucleotides which are able to fold into specific three-dimensional (3D) structures, generated using the Systematic Evolution of Ligands by EXponential Enrichment (SELEX) technique [7]. This process, relying on DNA or RNA libraries, is able to automatically synthesize a large variety of nucleic acid sequences with great selectivity for non-nucleic acid molecules. Aptamers are able to interact with their targets through structural recognition similar to that of the antibody-antigen reaction with a dissociation constant in the range of pico- to nano-molar. For these reasons, aptamers are usually referred as chemical Abs [8]. Moreover, an appealing characteristic of aptamers, as opposed to Abs, is their efficacy against small molecules where antibodies fail [9]. Different from Abs, the chemical nature of nucleic acids makes them easy to synthesize and engineer, and thereby, to obtain aptamers with extended bioavailability, regulating ability, and multi-functional properties [10,11]. Aptamers are thermally stable; even after denaturation temperature, they are able to refold into their 3D formations once at room temperature, in contrast to protein-based Abs, which totally lose their activity at high temperatures. The targets of aptamers range from small molecules to proteins, virus-infected cells, stem cells, and cancer cells. The chemical properties and biological activity of aptamers have made them attractive for use in biomedical applications ranging from bioassays to targeted therapy [12]. Moreover, aptamer technology has shown great potential for bioengineering of nanostructured devices. Aptamers used as bioprobes for the development of biosensors have heralded a new generation of biosensors called aptasensors [13]. A variety of aptasensors have been developed based on fluorescence, electrochemiluminescence, surface plasmon resonance (SPR) and surface-enhanced Raman scattering (SERS) [14,15,16]. Label-free optical aptasensors can be designed using porous silicon (PSi)-based devices. PSi is a nanostructured material which is widely used as a transducer surface in biosensing. Its sponge-like morphology, characterized by a specific surface area of about 500 m^2^ cm^−3^, ensures active and rapid interaction with the species to be detected [17]. Additional advantages of PSi are compatibility with semiconductor processing and largely tunable pore size (nanometers to microns), which makes possible the infiltration of appropriate-sized target molecules while excluding larger-sized, non-specific ones [18]. The result of PSi optical transducer response to binding of inorganic/organic matter on pore walls is a change of its average refractive index [19]. A PSi layer acts optically as a Fabry-Perot interferometer: the substitution of air in the pores enhances the average refractive index, resulting in a change in the reflectivity spectrum [20]. The analysis of the optical spectra by fast Fourier transform (FFT) could be used as a simple method to assess variations in the refractive index [21]. This review presents recent progress in the development of label-free PSi optical aptasensors for biomedical applications. Different PSi functionalization strategies for the development of such devices for the detection of molecules of diagnostic interest (i.e., insulin, bacteria, human thrombin) are described. In particular, the improvement of device performance in terms of sensitivity, response time and limit of detection (LoD) will be discussed.

## 2. Porous Silicon Optical Devices for Label-Free Biosensing

Over the past two decades, great interest has shown by many researchers in the improvement of label-free biosensors. Most standard biosensors need a label attached to the target, and their detection and quantification is assumed to correspond to the number of bound targets [22]. Labels are easily detectable entities, such as fluorophores, magnetic beads, active enzymes. However, the labeling procedure requires sample handling that can drastically change its biomolecule binding properties, as well as the target-label coupling reaction yield. In label-free biosensors, no label is required for the sensing. This technology is based on the direct measurement of a signal (optical, electrical or mechanical) which is generated by the interaction between the bioprobe and the analyte on the transducer surface [23,24,25]. Sensitive, fast, robust, low cost, label-free biosensors are highly desirable for a broad range of applications including medical disease monitoring, controlled release of drugs, and food security [26,27,28]. PSi has received remarkable interest as a transducer surface for the construction of low-cost, sensitive and biocompatible optical label-free biosensors [2]. This is mainly due to its intriguing physicochemical properties which makes possible the design of compact biosensors with high performance [29]. Moreover, PSi transducers can be optimized (pore size, pore depth, and porosity) to suit specific applications by controlling the etching parameters [30,31]. Label-free PSi optical biosensors, using interferometric reflectance spectroscopy (IRS), have demonstrated outstanding performance in terms of the rapid and reliable detection of several analytes [32]. IRS technique is based on a very simple set up: an incident white light is reflected on the two interfaces (air-PSi and PSi-bulk Si) of the porous material, producing a Fabry-Perot interference fringe pattern, depending on the optical thickness (physical thickness L times the refractive index (n) of the porous material) [33,34]. The fringe pattern is described by Equation (1);

(1) mλ = 2 nL

where λ is the maximum wavelength of two consecutive fringes with an order of magnitude *m*, L is the thickness of the porous layer and 2 nL is the effective optical thickness (EOT). The EOT can be determined by applying FFT to the observed interference fringe pattern; this parameter is usually used to measure the sensor response. When an analyte is captured by the bioprobe-modified PSi, a redshift of the fringe pattern is observed. This phenomenon is due to the substitution of air within the pores with the analyte—which has a higher refractive index—resulting in an increase in the EOT value [35]. Thus, the interferometric reflectance spectroscopy enables the simple detection of analytes by monitoring changes of EOT over time.

### Porous Silicon Fabrication and Surface Modification Strategies

The peculiar morphological, physical, and chemical properties of PSi make it one of the most explored nanostructured materials, as evidenced by the great number of papers about its features, and the prevalence of devices based on this material [36,37,38,39,40]. One reason for its success is the easy fabrication process based on a computer-controlled electrochemical etching procedure and a simple power supply. The PSi structure is obtained by electrochemical dissolution of doped crystalline silicon wafers in a hydrofluoric acid (HF) -based solution. Modulating parameters such as current density, type and concentration of crystalline silicon dopant, and the composition of electrolyte solution makes it possible to obtain porous structures with specific morphological and optical properties [31,39]. The silicon hydride (Si–H) terminated pore walls of as-etched PSi are prone to oxidation and dissolution under ambient conditions, such as atmospheric oxygen, water, and aqueous solutions [41]. The oxidation of PSi causes a significant change in the refractive index of the material (*n* = 3.5 for silicon, *n* = 1.4 for silicon dioxide), interfering with transduction signal of PSi optical biosensors. Moreover, dissolution in aqueous buffers leads to even greater changes in the refractive index (*n* = 3.5 for silicon, *n* = 1.33 for water), with a loss of signal due to PSi structural collapse [42]. The PSi surface should be properly stabilized for biosensing applications. A common method to prevent PSi from degradation is to intentionally grow an oxide layer on the surface via thermal oxidation, which reduces or completely removes the Si–H from the entire skeleton, substituting it for SiO_2_, which isotropically grows also in the pores [43]. To provide greater stability and protection against dissolution, the oxidized surface could be chemically modified with alkyl silanes. The two most popular silane coupling agents are 3-aminopropyl-triethoxysilane (APTES) and 3-aminopropyl-dimethyl-ethoxysilane (APDMES), both of which are able to form a dense monolayer on the PSi surface through Si–O–Si covalent bonds that limit the access of water to the underlying surface [44]. The hydrosilylation is an alternative surface-chemistry process involving the grafting of alkenes (or alkynes) to the hydride-terminated PSi surface, resulting in the formation of a monolayer of alkyl chains which is covalently attached to the surface through Si-C, showing much greater resistance to attack by nucleophiles such as water or hydroxide [41,45,46]. This reacting mechanism can be promoted by heat, light, Lewis acid catalysts in an inert atmosphere and completely deoxygenated/dried reagents, thus avoiding the formation of silicon oxides during the monolayer formation [47]. The grafting of alkyl silanes and alkanes/alkynes on PSi makes the surface chemically stable in aqueous solution, thereby avoiding surface oxidation or chemical degradation. Moreover, these passivation methods are valid strategies to functionalize the surface with reactive groups (−NH_2_, −COOH, −SH, and −CHO) for the subsequent conjugation of biomolecules (i.e., enzymes, proteins, Abs, peptides, DNA, aptamer). Additional methods used for PSi surface stabilization can be found in ref. [48]. From a sensing point of view, passivation/functionalization methods avoid the zero-point drift without altering the intrinsic sensitivity of the devices.

PSi biosensors are usually prepared by immobilizing the bioprobe on the transducer surface, once it has been synthesized by an ex situ procedure. However, there is also an innovative procedure for the preparation of biosensors based on the direct growth of the bioprobe (i.e., by in situ synthesis) on PSi used as support in so-called solid phase synthesis [49,50].

## 3. Label-Free Porous Silicon Apatasensor for Human Diseases Diagnosis 

Aptamers are an emerging class of single-stranded oligonucleotides which is generated using SELEX technology [8,51]. By folding them into well-defined secondary or tertiary structures, aptamers are able to specifically bind their target molecules with high affinity, and so are classified as powerful ligands for diagnostic and therapeutic applications [52]. They present significant advantages over conventional bioprobes (e.g., Abs), such as relatively low-molecular-weight and high stability, great affinity due to the remarkable low dissociation constants (K_d_) aptamer/target ranging from picomolar to nanomolar levels, and great selectivity thanks to their ability to recognize even minor structural differences between targets and their analogs. To date, aptamer-based biosensors have been successfully used for the detection of a large number of analytes of interest due to the highly selective interactions between the aptamer and the target, and the high amplification thanks to the optical, electrical or magnetic properties of the various sensing platforms. These novel integrations highlight the potential of aptamers as emerging tools for the fabrication of new sensing devices for the selective and sensitive detection of a wide range of targets, promising great advances in healthcare applications.

### 3.1. Aptamer-Decorated Porous Silicon Biosensor for Rapid Detection of Bacteria

**Figure 1:**
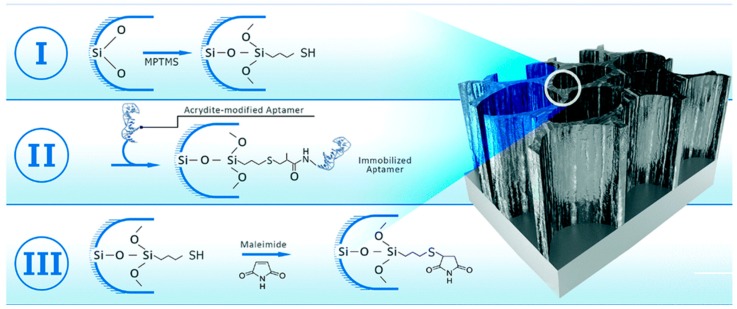
Three-step biofunctionalization process for aptamers immobilization onto PSiO_2_ device. (**I**) Silanization with MPTMS via a thioether bond, (**II**) reaction with acrydite-modified Hemag1P aptamer via a thioether bond and (**III**) blocking of residual thiol groups with maleimide. Reproduced with permission from [56].

**Figure 2:**
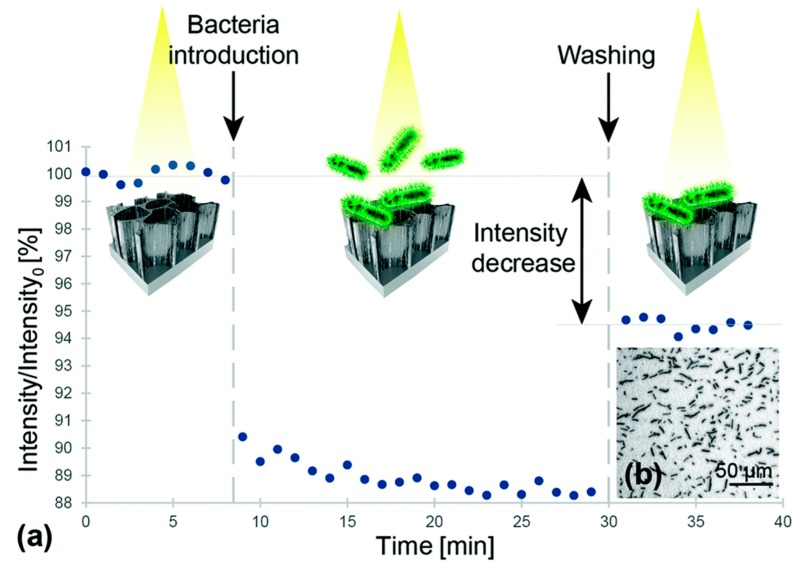
(**a**) Relative intensity change of the Hemag1P-modified PSiO_2_ device upon exposure to *L. acidophilus* bacterial suspensions (10^7^ cells per mL). (**b**) Microscope image taken after the biosensing experiment depicts *L. acidophilus* cells captured onto the aptamer-modified PSiO_2_ device. Reproduced with permission from [56].

Rapid detection and identification of bacterial contaminations in blood are a major challenge in today’s medical practice [53]. A bacterial contamination can occur in every environment, causing a specific disease in a variety of ways; even today, microbial diseases are a major cause of death in many countries [54]. The detection and identification of bacterial contaminations is still based on traditional microbiological techniques, which typically require several days to obtain results, making ‘real-time’ assessments unfeasible. Over the past decade, great efforts have been directed toward the development of new bioassays and biosensors for the rapid detection of pathogenic bacteria [55]. Various biosensors for fast bacteria detection have been reported; among them, the most popular are optical biosensors. These biosensors offer several advantages, including selectivity, speed, sensitivity, and reproducibility of measurements. Recently, Prof. E. Segal and co-workers at Technion, Haifa, Israel [56], described the design of an optical aptamer-based PSi biosensor for the direct capture of *Lactobacillus acidophilus*, employing the Hemag1P aptamer as a capture probe. The Hemag1P aptamer is 78-nucleotide-long sequence selected by the SELEX technique against the *L. acidophilus* membrane, i.e., a strain of bacteria which is important for the functional food and pharmaceutical industry. This aptamer is able to target the S-proteins which are abundantly present on the bacteria membrane. The first step in the Hemag1P-PSi aptasensor preparation was the anodization process of a Si wafer (300 mA cm^−2^, 30 s) and its thermal oxidation, thus obtaining oxidized PSi (PSiO_2_). The PSiO_2_ is decorated with the aptamer through the three-step biofunctionalization route, as illustrated in Figure 1. The PSiO_2_ is first silanized with 3-mercaptopropyl-trimethoxysilane (MPTMS) and then reacted with acrydite-modified Hemag1P aptamer via a thioether bond. The final functionalization step is the blocking of residual thiol groups of the aptamer with maleimide, in order to reduce non-specific reactions with the buffers. The success of aptamer immobilization on the PSi surface was confirmed using attenuated total reflectance Fourier transform infrared (ATR-FTIR) spectroscopy and Ellman colorimetric assay for thiol-groups. The biosensing mechanism of the Hemag1P-aptasensor was based on monitoring changes in the intensity of fast Fourier transformation (FFT) peaks, obtained from the raw PSi reflectivity spectra during exposure to bacteria suspensions. The Hemag1P-modified PSiO_2_ biosensor was exposed to *L. acidophilus* suspensions while the reflectivity spectra changes of the device were monitored in real time (Figure 2). Firstly, the initial intensity baseline was established by exposing the aptasensor to the aptamer selection buffer solution (SB). Then, the biosensor was incubated with the bacteria solution (10^7^ cells per mL in SB) for 20 min, allowing bacteria/aptasensor interactions to occur. Consequent washing of the biosensor with SB was performed to remove unbound bacteria, after which the intensity increased and stabilized at a net intensity decrease value of 5.5%. Several replications of this biosensing experiment have demonstrated similar behavior to that represented in Figure 2, and a highly reproducible net intensity decrease value of 5.5% (0.07%), confirming the ability of the biosensor to detect 10^6^ cells per mL of *L. acidophilus*.

**Figure 3:**
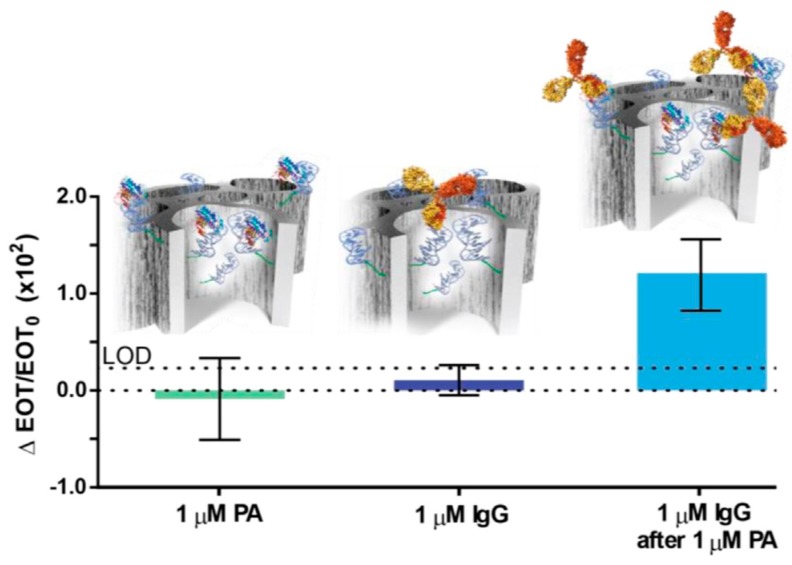
Sensitivity enhancement of the PAA-functionalized PSiO_2_ biosensor. Averaged optical response (relative EOT) of the biosensor upon exposure to 1 M of: PA, IgG or both in a successive manner. Schematic illustration of biomolecules captured by the aptamers within the porous scaffold. Upper dashed line indicates the LoD value. Differences between both single exposures (of PA or IgG) and the sandwich assay are statistically significant (*p* < 0.05, *n* ≥ 3). Reproduced with permission from [58].

*Staphylococcus aureus* is a major cause of bacteremia and infections in humans, as well as food-borne diseases [57]. *S. aureus* treatment is especially challenging due to the bacteria’s ability to rapidly adapt and develop resistance to antibiotics; thus, a fast and reliable means of detection of such bacteria is crucial for the effective control of infection. Protein A (PA, 45 kDa), secreted by and displayed on the cell membrane of *S. aureus*, is considered a significant biomarker for this bacteria. K. Urman et al. [58] developed a label-free optical PSi aptasensor for the specific detection of PA. Protein A-targeting aptamers (PAA) are conjugated to APTES-modified PSiO_2_ thin films by standard carbodiimide-coupling chemistry [59,60]. PSiO_2_ surface modification and aptamer-conjugation were confirmed by ATR-FTIR analyses. The PAA-PSiO_2_ biosensor was exposed to PA solutions at different concentrations, and the biosensor response, evaluated as effective optical thickness (EOT) changes, showed a relative EOT increment with increasing PA concentration. The results demonstrated a specific detection and quantification of PA in a range of 2–50 μM, with a binding affinity towards PA of 13.98 μM and LoD of 3.17 μM. Due to the affinity between PA and the antibody, IgG was introduced in a sandwich-assay format to enhance the sensitivity of the biosensor by three fold. In Figure 3, the exposure of a PAA-biosensor to either PA or IgG (as control) gave insignificant signals, i.e., below the critical value (3 × σ = 0.205) calculated for the LoD. The values obtained by the sandwich assay were six times higher (1.2 ± 0.3 as ΔEOT/EOT_0_ × 10^3^), resulting in a PA LoD value of 1 μM. This work demonstrated a proof-of-concept scheme for increasing three-fold the sensitivity of PAA-functionalized PSi biosensors by taking advantage of PA and IgG affinity.

### 3.2. Porous Silicon Aptasensor for Detection of Insulin 

**Figure 4:**
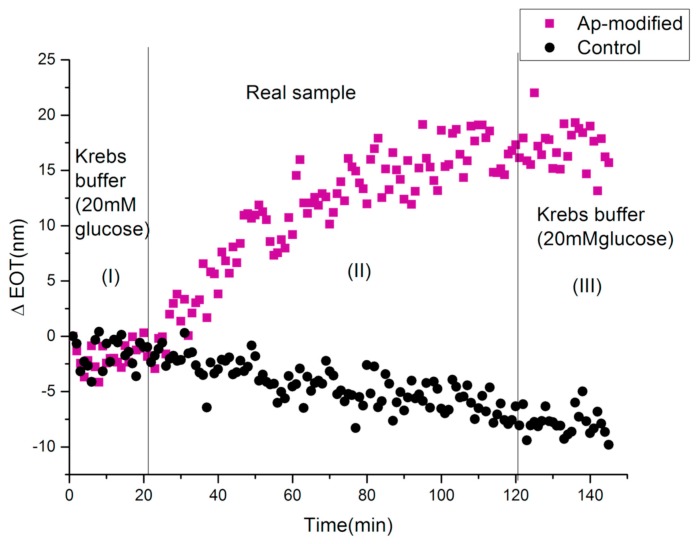
Sensorgram for the detection of insulin secreted by human islets upon stimulation with 20 mM glucose for 2 h in Krebs buffer using IRS. Reproduced with permission from [63].

Diabetes mellitus is a worldwide health problem, and severe complications associated with this disease are causes of death [61]. Diabetes mellitus is a pathological state resulting from an absolute or relative deficiency of insulin in the body. Insulin is a hormone synthesized by the β cells of the pancreatic Islets of Langerhans. The carrier glucose transporter (type 4) is able to bind with insulin, allowing glucose entry to the heart, muscles, and brain cells. When the regulatory mechanisms fail, a hyperglycemia occurs; blood glucose concentrations increase to over 7.0 millimoles per liter, and random glucose concentrations increase to over 11.1 millimoles per liter [62]. Long-term hyperglycemia may be the cause of several life-threatening complications such as cardiovascular diseases, diabetic nephropathy, neuropathy, and retinopathy. The monitoring of glucose blood levels is the first step in diagnoses of diabetes mellitus, whereby a rapid and accurate diagnosis method is necessary for the prevention of lethal complications. Standard diagnostic techniques (e.g., enzyme-linked immunosorbent assay (ELISA), radioimmunoassay (RIA), chromatography) are typically laborious, expensive, time-consuming and require sophisticated laboratory equipment. Recently, Prof. N.H. Voelker and co-workers at Monash Institute, Melbourne, Australia [63], developed two different label-free PSi-based optical biosensors for the detection of insulin secreted by human pancreatic islets: an antibody-modified PSi (Ab-modified) and an aptamer-modified PSi (Ap-modified). Freshly etched PSi single layer structures were exposed to a thermal hydrosilylation reaction with undecylenic acid, followed by reaction with carbodiimide chemistry (EDC/NHS), the immobilization of the Ab (150 kDa) or the aptamer (9.7 kDa) and, finally, (d) quenching of active NHS ester with bis-PEG-amine (BPA). Both of the prepared PSi devices (i.e., Ab-modified- and Ap-modified-PSi) were tested as optical biosensors for the detection of insulin under the same conditions in a Krebs Ringer buffer (a solution used for glucose-stimulated insulin secretion clinical assay). The devices were exposed to Krebs buffer for 20 min to establish a baseline, and no significant change in ΔEOT value was observed. The exposure of the PSi devices to insulin (50 μg/mL) solution for 60 min increased ΔEOT, and finally, rinsing with Krebs buffer for 20 min was carried out. The results showed that Ap-modified PSi outperformed Ab-modified PSi devices in terms of insulin detection time and sensitivity: 19 nm EOT shift in 12 min compared to 16 nm EOT shift in 60 min. The rapid response of Ap-modified PSi is associated with the exclusive conformation change of the aptamer during insulin binding, highlighting the potential benefit of using aptamers rather than Abs as bioprobes in biosensor development. Ap-modified PSi was tested as a biosensor in a real sample detection of insulin secreted by human donor islets. Human islets (40,000 IEQ) were stimulated with 20 mM glucose for 2 h in Krebs buffer, after which the cells were centrifuged and the insulin-containing supernatant was used for the sensing experiment, following the previously-described protocol. The exposure of the Ap-biosensor to insulin (20 mM) in Krebs solution showed a gradual increase in ΔEOT with a maximum shift of ~17 nm over 80 min (Figure 4). After the washing step with Krebs buffer, no significant change in ΔEOT was observed. Applying the maximum ΔEOT shift to the calibration curve obtained for the Ap-modified surface, a concentration of 15.4 μg/mL was obtained. In addition, no significant changes to ΔEOT were observed on the control surface, except for a small blue shift as a result of a slight degradation of PSi in the aqueous medium. For comparison, the ELISA technique was used to determine the level of insulin in the real sample, showing a result of 16.9 ± 0.2 μg/mL, which is in agreement with the result of the Ap-PSi biosensor. These results demonstrated, for the first time, the great capability of the Ap-modified PSi surface to detect insulin secreted by human islets from a donor upon stimulation with glucose.

**Figure 5:**
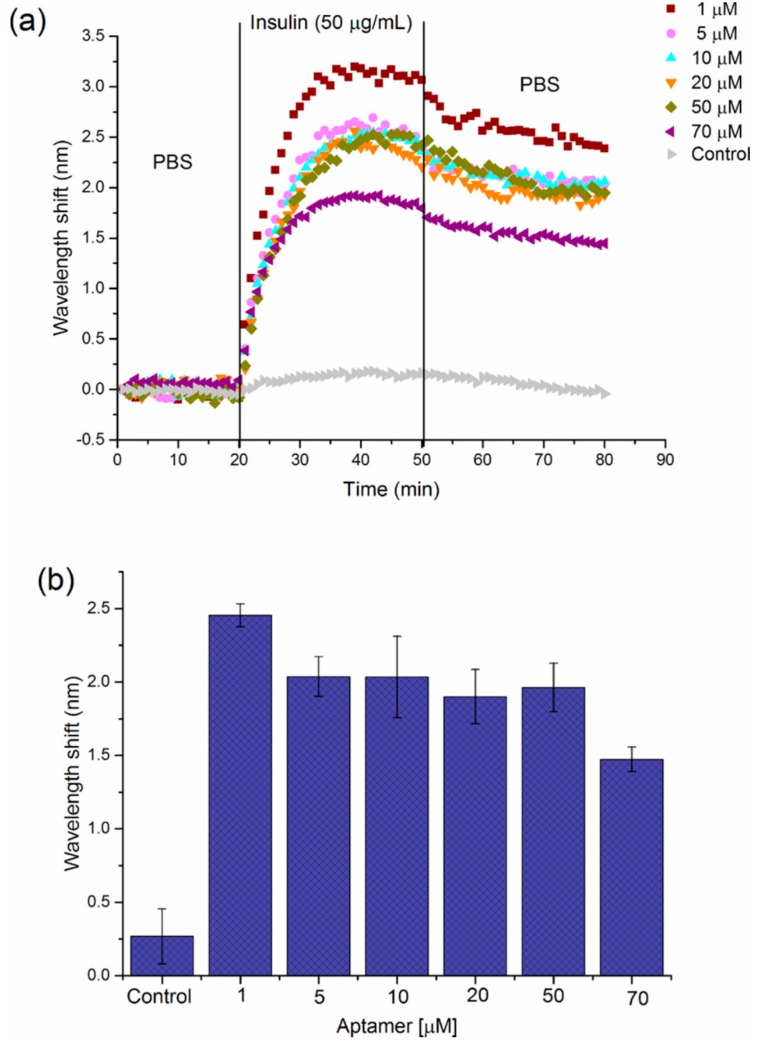
(**a**) Sensograms showing the effect of IBA solution concentration used for modified-PSi device on the response to 50 μg/mL insulin. (**b**) Graph representing the average wavelength shift (nm) obtained for detection of 50 μg/mL insulin by PSi-5 cycles surface, which is modified with a range of aptamer concentrations (1–70 μM). Error bars correspond to the standard deviation from three individual experiments on PSi-devices prepared with each IBA concentration. Reproduced with permission from [64].

Recently, N.H. Voelker and co-workers [64] demonstrated the optimization of PSi fabrication for the development of optical insulin biosensors with high sensing performance in terms of response time and LoD. PSi rugate filter (PSiRF) were modified by thermal hydrosilylation and conjugated to an insulin binding aptamer (IBA) at different concentrations (1, 10, 20, 50 and 70 μM) via carbodiimide chemistry. The different prepared surfaces were tested for insulin detection (50 μg/mL) using IRS in order to verify the effect of IBA concentration on biosensing device performance. Insulin biosensing was carried out on each prepared PSi device with 20 min PBS baseline, 30 min 50 μg/mL insulin flow, and 30 min PBS washing. Generally, a high bioprobe density is preferred in biosensor development in order to guarantee the highest target binding capacity. In this specific case, the obtained result showed the opposite trend, i.e., the surface obtained with a low aptamer concentration (1 μM) performed better compared to the surface prepared using the highest aptamer concentration (70 μM) (Figure 5).

This phenomenon might be due to a steric hindrance effect that could prevent the aptamer from folding into the correct 3D conformation required to show the highest insulin affinity [64]. The optimized PSi biosensor platform was then applied to detect insulin in a real sample. For biosensing experiments with IRS, the sensor surface was exposed to Krebs buffer flow for 20 min, followed by supernatant containing the insulin secreted by human islets (20000 IEQ), and then washed with Krebs buffer for 30 min. At first, a steady baseline was achieved in the Krebs buffer, and no shift in the peak wavelength was observed. Then, the supernatant with secreted insulin was flowed over the biosensor surface and a gradual red shift was observed, as a result of insulin/IBA binding. During the final washing step, a decrease in the maximum wavelength was observed due to the removal of unbound insulin from the biosensor surface. A shift of 0.19 nm calculated by subtracting the final wavelength value from the initial baseline value was applied to the linear regression equation from the calibration curve (data not shown) in Krebs buffer and the calculated concentration of insulin in the real sample was 1.3 μg/mL. This result was confirmed by an ELISA test validating the PSiRF sensor as a successful device for the detection of insulin secreted by human islets stimulated with glucose.

### 3.3. Macroporous Silica Aptasensor for Label-Free Optical Quantification of Human Thrombin

**Figure 6:**
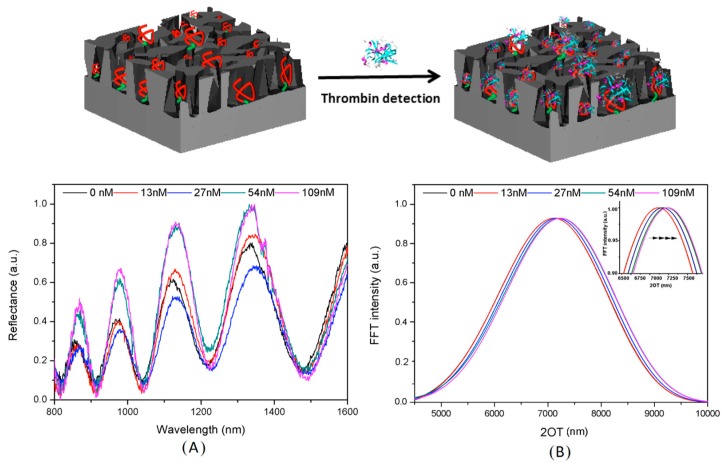
Reflectivity spectra (**A**) and corresponding Fourier transforms (**B**) of PSi-aptasensor after exposure to different thrombin concentrations (13, 27, 54 and 109 nM). Reproduced with permission from [71].

Human thrombin (MW ≈ 37 kDa) is a serine protease—also known as coagulation factor II—which is able to convert soluble fibrinogen (factor I) into insoluble strands of fibrin (factor Ia), with a fundamental role in coagulation and hemostasis. The balance between its production and inhibition avoids hemorrhagic or thrombosis phenomena, which may be fatal to human health. In a healthy subject, thrombin concentration is almost absent until it passes from from nM to µM levels during the coagulation process [65]. Pathological coagulation disorders, such as ischemic stroke or thromboembolism, could be induced by high levels of thrombin in the blood (beyond the normal coagulation phenomenon) [66]. Moreover, the deregulation of neuronal PAR1-activation by thrombin has been associated with many disorders of the central nervous system (SNC), including the Alzheimer and Parkinson diseases, and the role of thrombin in cancer is well known [67,68]. The important role of thrombin in different (patho)physiological processes has triggered interest in the possible discovery of novel thrombin inhibitors, as well as in the development of new devices which are capable of rapidly detecting its level in blood with high selectivity and very low LoD [69,70]. M. Terracciano et al. [71] described the fabrication of a label-free PSi optical aptasensor by in situ synthesis of a 15-mer thrombin binding aptamer (TBA) on silanized macro-PSi for the quantification of human α-thrombin levels. The high sensitivity, selectivity, and reversibility of the obtained aptasensor were also demonstrated. Macro-PSi structure (pores size > 50 nm), after thermal oxidation, was functionalized by grafting aminosilane compound (APTES), and bioconjugated to a TBA probe by an in situ synthesis [72]. Figure 6 shows the reflectivity spectra (A) with the corresponding FFTs (B) of APTES-modified PSi before and after the in situ synthesis process. The calculated FFT peak shift of 36 nm confirmed the success the TBA growth on the PSi structure. This result was also confirmed by the coupling yield analysis of the 5′-dimethoxytritil (DMT) group released into the solution after each synthesis cycle using ultraviolet (UV) spectroscopy. The PSi surface functionalization with TBA (F_TBA_) was quantified using the Lambert-Beer law (DMT molar absorptivity ε = 71,700 M^−1^ cm^−1^, λ = 500 nm) and UV intensity value corresponding to the last synthesized nucleotide, i.e., I_N17_ = 0.055 ± 0.001. The F_TBA_ was calculated to be (1.92 ± 0.03) × 10^–5^ mol/g for PSi sample with a weight of 0.2 mg. The PSi surface functionalization in terms of nmol cm^–2^ evaluated using the ratio F_TBA_/SSA (specific surface area of PSi 199 m^2^ g^−1^ evaluated by the Brunauer–Emmett–Teller analysis) was found to be 0.0125 ± 0.0002 nmol cm^–2^. For biosensing experiments, the aptasenosr was exposed to different thrombin concentrations (13, 27, 54 and 109 nM). The TBA-thrombin interaction was monitored using spectroscopic reflectometry, demonstrating the technique’s ability to recognize the analyte at different molar concentrations. The calculated affinity constant was 14 ± 8 nM, with a sensitivity of 4.1 ± 0.8 nm nM^−1^ and LoD of 1.5 ± 0.3 nM. The LoD value was lower than other well-known, very sensitive assays [73]. Moreover, the reversibility of the PSi-aptasensor was also proved. The PSi-aptasensor was regenerated by treatment in water at 53 °C, corresponding to the melting temperature of TBA [74]. At this temperature, TBA lost the typical G-quadruplex structure, as well as its affinity with thrombin. However, this process was reversible: by exposing TBA-PSi to thrombin and/or monovalent cations at room temperature, the aptamer folded again into a G-quadruplex structure [75]. The device regeneration induced a decrease of optical thickness (about 14 nm) due to the release of thrombin from the PSi pores. An increase of 38 nm was observed after exposing the device to 109 nM of thrombin solution, thus proving the ability of the aptasensor to recognize the analyte. The results reported in this work endorse macro–PSi as a suitable substrate for the realization of a wide range of aptasensors using an in situ synthesis approach for surface functionalization.

## 4. Conclusions

Label-free optical PSi biosensors using aptamers as bioprobes represent a promising class of devices in future healthcare diagnoses. Aptamers have emerged as new molecular recognition tools with great affinities and specificities. The integration and automation of aptamers are easier than conventional bioprobes, and are extremely convenient for the development of biosensors. PSi is an appealing nanostructured material which is largely used in biosensing due to the ease with which its properties, e.g., pore morphology, photonic properties, biocompatibility and surface chemistry, may be tuned.

In this review article, we summarized the recent progress in the development of label-free optical PSi-aptasensors for human diseases diagnosis such as bacterial infections, real-time monitoring of insulin and human α-thrombin. Particular attention was paid to PSi fabrication and functionalization strategies; it was shown that PSi-aptasensors demonstrate high levels of performance in terms of stability, sensitivity, early detection and reversibility. Morover, the LoD values of the described PSi optical aptasensor were found to be comparable or below those of other well-known electrical or electrochemical aptasensors. Moreover, optical devices are preferred because opto-instruments are non-invasive and safe, even in harsh conditions such as in vivo monitoring inside a patient’s body, where, for example, electrical devices could be harmful. The potential of aptasensors appears to be huge, and this exciting area is seeing exponential growth. The capacity to develop high affinity-based detection systems with tailored characteristics offers to the biosensing field the chance to explore new and dynamic routes of biosensor development. The possibility of commercializing aptasensors in the near future can therefore be reasonably foreseen.
